# Transfusion practice in the non-bleeding critically ill: an international online survey—the TRACE survey

**DOI:** 10.1186/s13054-019-2591-6

**Published:** 2019-09-11

**Authors:** Sanne de Bruin, Thomas W. L. Scheeren, Jan Bakker, Robin van Bruggen, Alexander P. J. Vlaar, on behalf of the Cardiovascular Dynamics Section and Transfusion Guideline Task Force of the ESICM, Massimo Antonelli, Cecile Aubron, Maurizio Cecconi, Joanna C. Dionne, Jacques Duranteau, Nicole P. Juffermans, Dirk de Korte, Jens Meier, Gavin Murphy, Simon Oczkowski, Anders Perner, Timothy Walsh

**Affiliations:** 1 Department of Intensive Care Medicine, Amsterdam University Medical Centers, University of Amsterdam, Room C3-430, Meibergdreef 9, 1105 AZ Amsterdam, The Netherlands; 2 Department of Blood Cell Research, Sanquin Research, and Landsteiner Laboratory, University of Amsterdam, Amsterdam, The Netherlands; 3 Department of Anaesthesiology, University Medical Centre Groningen, University of Groningen, Groningen, The Netherlands; 4 Department of Intensive Care Medicine, Erasmus MC University Medical Center, Erasmus University Rotterdam, Rotterdam, The Netherlands; 5 Department of Intensive Care Medicine, New York University Medical Center and Columbia University Medical Center New York, New York City, USA; 6 Ponfificia Universidad Católica de Chile, Santiago, Chile

**Keywords:** Transfusion practice, Critically ill, Red blood cells, Platelets, Plasma

## Abstract

**Background:**

Over the last decade, multiple large randomized controlled trials have studied alternative transfusion strategies in critically ill patients, demonstrating the safety of restrictive transfusion strategies. Due to the lack of international guidelines specific for the intensive care unit (ICU), we hypothesized that a large heterogeneity in transfusion practice in this patient population exists. The aims of this study were to describe the current transfusion practices and identify the knowledge gaps.

**Methods:**

An online, anonymous, worldwide survey among ICU physicians was performed evaluating red blood cell, platelet and plasma transfusion practices. Furthermore, the presence of a hospital- or ICU-specific transfusion guideline was asked. Only completed surveys were analysed.

**Results:**

Nine hundred forty-seven respondents filled in the survey of which 725 could be analysed. Hospital transfusion protocol available in their ICU was reported by 53% of the respondents. Only 29% of respondents used an ICU-specific transfusion guideline. The reported haemoglobin (Hb) threshold for the general ICU population was 7 g/dL (7–7). The highest reported variation in transfusion threshold was in patients on extracorporeal membrane oxygenation or with brain injury (8 g/dL (7.0–9.0)). Platelets were transfused at a median count of 20 × 10^9^ cells/L IQR (10–25) in asymptomatic patients, but at a higher count prior to invasive procedures (*p* < 0.001). In patients with an international normalized ratio (INR) > 3, 43% and 57% of the respondents would consider plasma transfusion without any upcoming procedures or prior to a planned invasive procedure, respectively. Finally, doctors with base specialty in anaesthesiology transfused critically ill patients more liberally compared to internal medicine physicians.

**Conclusion:**

Red blood cell transfusion practice for the general ICU population is restrictive, while for different subpopulations, higher Hb thresholds are applied. Furthermore, practice in plasma and platelet transfusion is heterogeneous, and local transfusion guidelines are lacking in the majority of the ICUs.

**Electronic supplementary material:**

The online version of this article (10.1186/s13054-019-2591-6) contains supplementary material, which is available to authorized users.

## Introduction

As critically ill patients frequently develop anaemia, thrombocytopenia or coagulopathy [1–3], transfusion of blood components is a frequent intervention in the intensive care unit (ICU). About 12.5% of all transfused red cell concentrates (RCCs), 13% of all platelet concentrates (PC) and 30% of all plasma units in the hospital are transfused in the ICU [4]. However, these products are associated with life-threatening adverse events including transfusion-related acute lung injury (TRALI), transfusion-associated cardiac overload (TACO) and transfusion-related immunomodulation (TRIM) [5–7].

Since the Transfusion Requirements in Critical Care (TRICC) trial, 20 years ago, it has been increasingly recognized that a restrictive RCC transfusion strategy may be as safe as a liberal strategy and even reduce patient mortality in specific patient subpopulations [8]. Consequently, ICU transfusion practice has shifted towards more restrictive strategies. From 2002 to 2012, the incidence of RCC transfusion in critically ill patients has dropped from 37 [9] to 26% [1] during ICU admission. This reduction coincided with the publication of multiple large international randomized controlled trials (RCTs) showing the safety of a restrictive transfusion strategy [8, 10, 11].

While multiple large RCTs have been performed to compare liberal versus restrictive strategies in red blood cell transfusions in ICUs, RCTs studying the optimal transfusion strategies in critically ill patients for plasma and platelets are limited or had a small sample size [12]. It is difficult to judge what “appropriate” transfusion triggers are for these blood products. This uncertainty is reflected in poor adherence to recommended best practices. It is estimated that hospital-wide 37% of transfused plasma units and 33% of transfused platelets are administered outside the guideline recommendations [13–15].

Of note, there is no international ICU transfusion guideline. The aim of this survey was to evaluate the use of local transfusion guidelines in the ICU and the applied transfusion thresholds for RCC, platelet (PLT) and plasma transfusion in ICU patients without an active haemorrhage.

## Methods

### Survey

An anonymous survey on transfusion practices in non-bleeding patients was conducted among intensivists, intensivist in training and non-intensivist specialists attending in the intensive care medicine. This survey was initiated by the Cardiovascular Dynamics Section and endorsed by the European Society of Intensive Care Medicine (ESICM). In addition, multiple national intensive care societies distributed the survey to its members by newsletters and/or promoted it on their website (see Additional file 1 for contributors).

### Study design

An online platform was used to set up the questionnaire (SurveyMonkey, Portland, OR, USA). After designing, the survey was tested by an international panel of intensivists to optimize the validity and accuracy of the questionnaire. The survey included 40 questions, divided into 4 sections: respondent demographics, transfusion practice regarding red blood cells (15–17 questions), platelets (5 questions) and plasma transfusions (8 questions, see Additional file 1 for static version). Multiple clinically relevant subpopulations (also non-bleeding) were addressed in each section. For red cell transfusion, first, the preferred haemoglobin (Hb) threshold for the general ICU population was asked, followed by the preferred Hb level for each subpopulation. For platelet transfusion, a distinction was made between transfusions prophylactically and prior to different invasive procedures. For plasma transfusions, a distinction was made between prophylactic transfusion without a planned procedure and prior to an invasive procedure in general.

### Statistical analysis

Only completed surveys were analysed. Since some questions were not applicable for all doctors, respondents were allowed to leave specific questions about subgroups/specific interventions open. This missing data was not imputed.

Descriptive statistics were used to characterize the respondent demographics. Normally distributed and non-normally distributed data were reported as mean (standard deviation) or as median (first quartile-third quartile), respectively. Categorical data was presented as percentage. Participants were able to fill in Hb thresholds in g/dL, g/L, or mmol/L, and all answers were converted to g/dL for analysis.

Transfusion thresholds were not normally distributed; therefore, the Kruskal-Wallis test was used to test whether the transfusion thresholds differed significantly in subpopulations or between interventions. As a post hoc test, the Dunn test with Bonferroni correction was used. In addition, transfusion thresholds were analysed using the Wilcoxon sum rank test or Kruskal-Wallis test to test the dependence of two grouping variables or more than two grouping variables, respectively. Chi-square test with Yates’s correction for continuity was used for categorical variables. For comparing different world regions, only the regions where at least ten respondents were working were taken into account because they may not accurately represent the transfusion practice across their region. All tests were two sided. A *p* value < 0.05 was considered to be statistically significant. Statistical tests were performed with R studio (2018, 3.5.1, Vienna).

## Results

### Demographics

The survey was open for 6 months (June 2018 to November 2018). Of the 947 received surveys, 769 were complete; of these, 44 were excluded because the respondents did not fulfil the study inclusion criteria (i.e. non-physician or paediatric ICU physician). The remaining 725 who completed the surveys (representing 69 countries) were included in the study. The majority of the participants practised ICU in Europe (76%) (Additional file 1: Figure S1). Background specialties were mainly anaesthesiology (62%) and internal medicine (20%); other demographics are shown in Table 1.

**Table 1:** Respondents demographics

Demographics	No. of respondents (%)
Certification level	
Intensivist	589 (81)
Resident, specialist in training	53 (7)
Specialist, non-intensivist practising ICU	73 (10)
Others	10 (1)
Primary medical specialty	
Anaesthesiology	450 (62)
Cardiology	18 (2)
Internal medicine	144 (20)
Neurology	4 (1)
Pulmonology	25 (3)
Surgery	15 (2)
Others	67 (10)
Type of intensive care unit (ICU)	
Medical ICU	63 (9)
Surgical ICU	536 (74)
Mixed ICU	110 (15)
Others	16 (2)
Number of ICU beds	
< 10	209 (29)
10–15	206 (28)
16–20	115 (16)
> 20	193 (27)
Not specified	2 (0)
Annual number of patients treated in the ICU	
< 500	178 (25)
501–1000	239 (33)
1001–1500	139 (19)
1501–2000	66 (9)
> 2000	98 (14)
Not specified	5 (1)
Type of institution	
University hospital	326 (45)
University-affiliated hospital	146 (20)
Non-university public hospital	183 (25)
Private hospital	64 (9)
Others	6 (1)
Which unit do you use to measure haemoglobin?	
g/dL	499 (69)
g/L	171 (24)
mmol/L	55 (8)

### Red cell transfusion

**Fig. 1:**
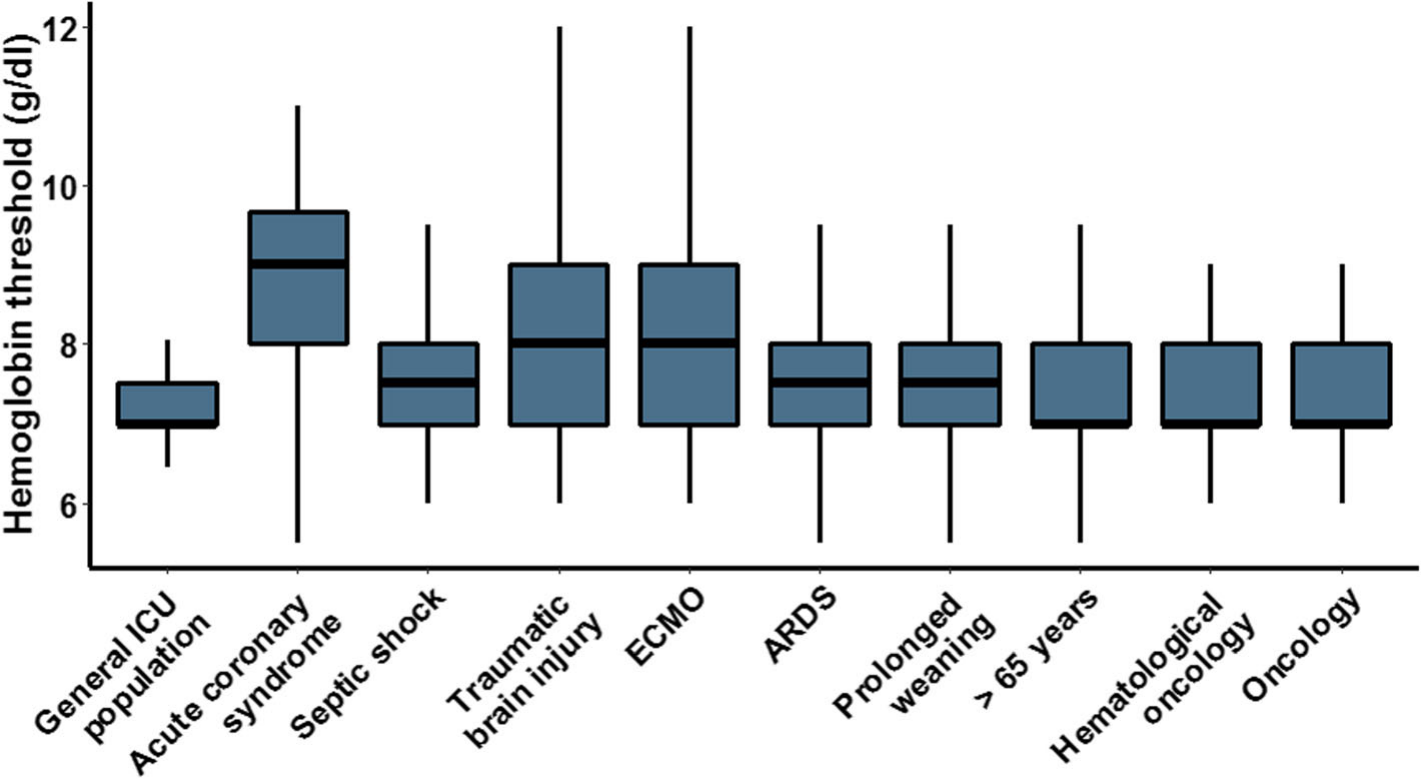
Respondents were asked which Hb threshold they used for RCC transfusion in the general ICU population and different subpopulations. Respondents used in the general population a Hb threshold of 7.0 g/dL (7.0–7.5). This is significantly lower (*p* < 0.001) compared to patients with acute coronary syndrome (9.0 g/dL (8–9.7)), septic shock (7.5 g/dL (7.0–8.0)), acute brain injury (8.0 g/dL (7.0–9.0)), patients undergoing ECMO (8.0 (7.0–9.0) g/dL), issues of prolonged weaning (7.5 g/dL (7.0–8.0)), or patients with ARDS (7.5 g/dL (7.0–8.0)). No statistical differences were observed between the general ICU population and patients older than 65 years and patients with (haematological) oncology (all three groups were transfused at a Hb threshold of 7.0 g/dL (7.0–7.5))

The median reported Hb threshold used in the general ICU population was 7 g/dL (7.0–7.5). Higher Hb transfusion thresholds were reported in patients with acute coronary syndrome (ACS), septic shock, acute brain injury, those receiving extracorporeal membrane oxygenation (ECMO), with acute respiratory distress syndrome (ARDS), age over 65 years and with prolonged weaning (*p* < 0.001 for all patient populations, see Fig. 1). Thresholds did not differ between oncological/haematologic patients and the general ICU population (*p* = 1). The largest variation in transfusion thresholds was observed in patients on ECMO and in patients with traumatic brain injury. Respondents would transfuse these patient populations at a Hb threshold of 8.0 g/dL (7.0–9.0). The highest Hb threshold was reported for patients with ACS median of 9.0 g/dL (8.0–9.7).

Following the transfusion of the first RCC, Hb levels were routinely not re-evaluated before transfusing a second unit. Of the respondents, 28% always re-evaluate the Hb level while 16% never re-evaluate.

### Transfusion triggers

**Fig. 2:**
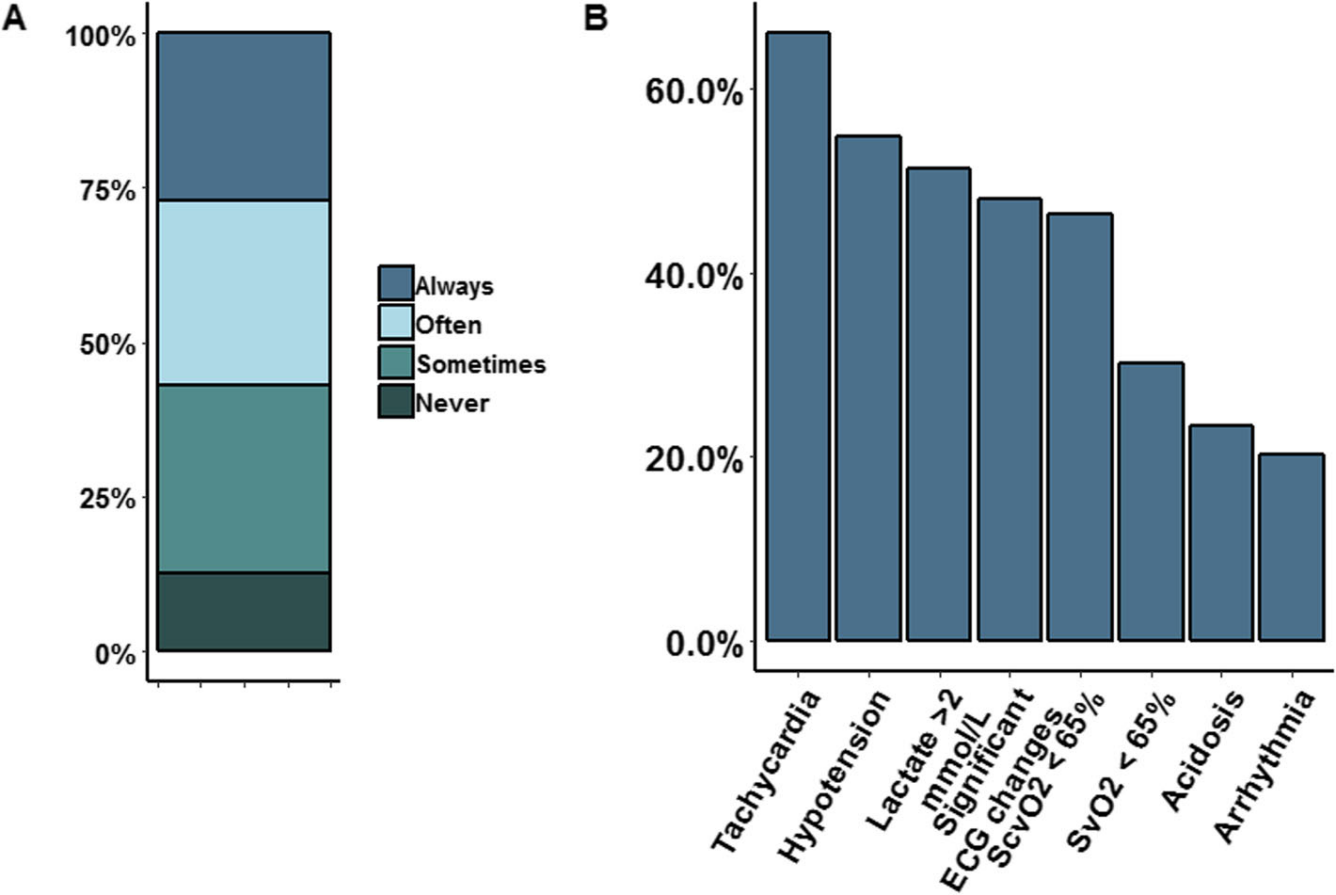
**a**, **b** The use of transfusion triggers in addition to a haemoglobin threshold

The majority of the respondents used clinical markers such as hypotension and tachycardia along with Hb levels to guide the transfusion. Among the respondents, only 13% never uses other physiological triggers in addition to a Hb threshold. Of interest, 27% of the respondents would always use other physiological triggers (Fig. 2a). Tachycardia (66%), hypotension (55%) and lactate levels > 2 mmol/L (51%) were mentioned most often (Fig. 2b), while significant ECG changes were ranked as the most important physiological trigger.

### Prevention of RCC transfusion

The use of iron or iron in combination with erythropoietin (EPO) to improve erythropoiesis and prevent RCC transfusion was reported by 41% and 17% of respondents, respectively. EPO was reported by 12% of the respondents as a monotherapy. A quarter of the respondents would never use these pharmacological agents for this purpose. Non-pharmacological blood conservation measures were less common in the ICU. Closed loop blood sampling was the most common intervention (23%), followed by microtube sampling (13%). Computerized decision support was used by only 2% of the respondents.

### Platelet transfusion

**Fig. 3:**
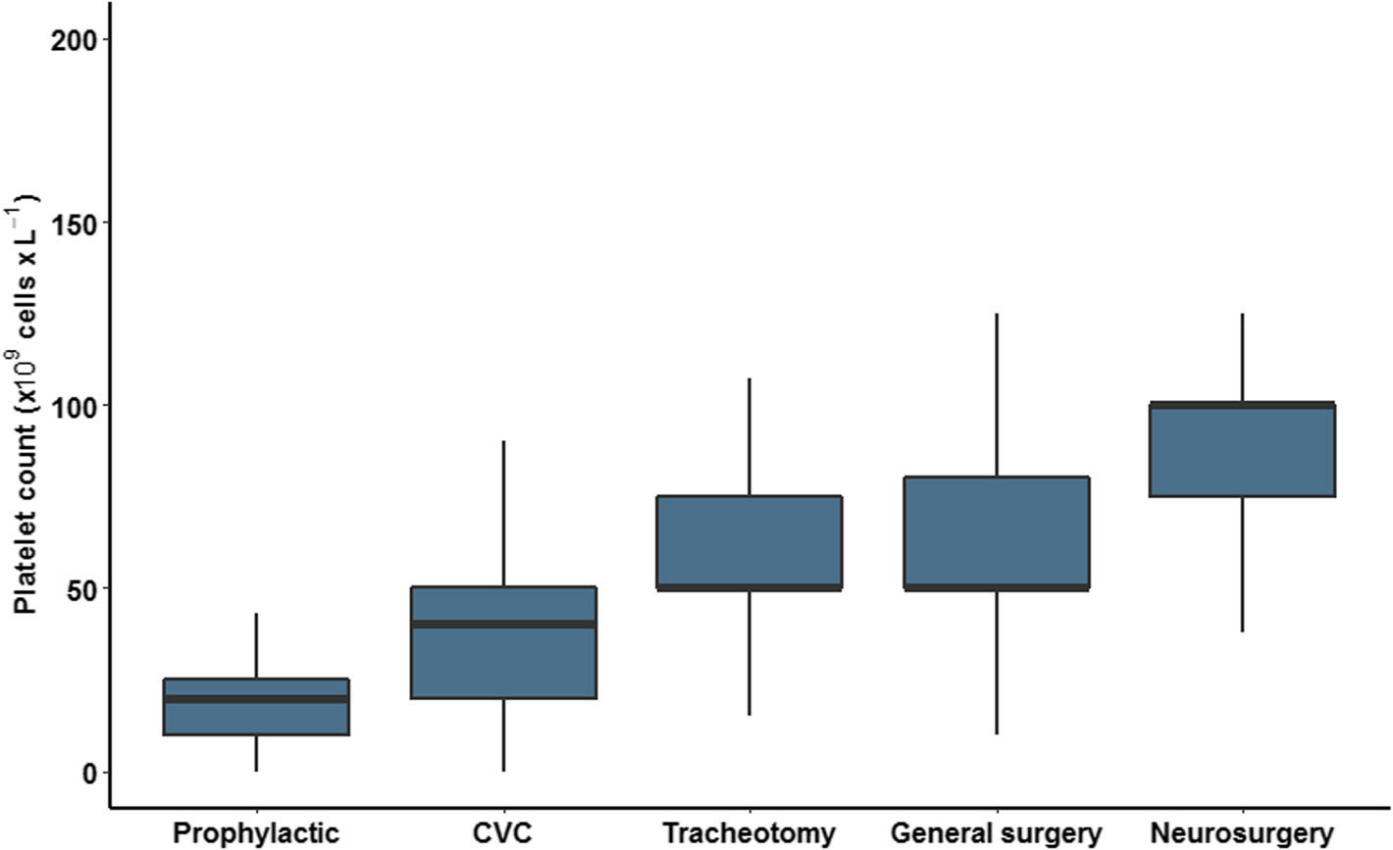
Prophylactic platelet thresholds without any planned invasive procedure and  prior to different procedures

In non-bleeding patients without a planned invasive procedure, respondents would transfuse patients at a platelet count of 20 × 10^9^ cells/L (10–25). Platelet concentrates were transfused at higher platelet counts prior to an invasive procedure (*p* < 0.001). Respondents would transfuse at a platelet count of 40 × 10^9^ cells/L (20–50) prior to central venous catheter (CVC) placement, 50 × 10^9^ cells/L (50–75) prior to tracheotomy, 50 × 10^9^ cells/L (50–80) prior to general surgery and 100 × 10^9^ cells/L (70–100) prior to neurosurgery (Fig. 3). When transfusing a PLT concentrate, 18% of the respondents never re-evaluate the platelet count before transfusing a second unit.

### Coagulopathy

The majority (87%) of the respondents transfuse their patients with fresh frozen plasma; only 9.5% reported to use pooled plasma to correct coagulopathy.

**Fig. 4:**
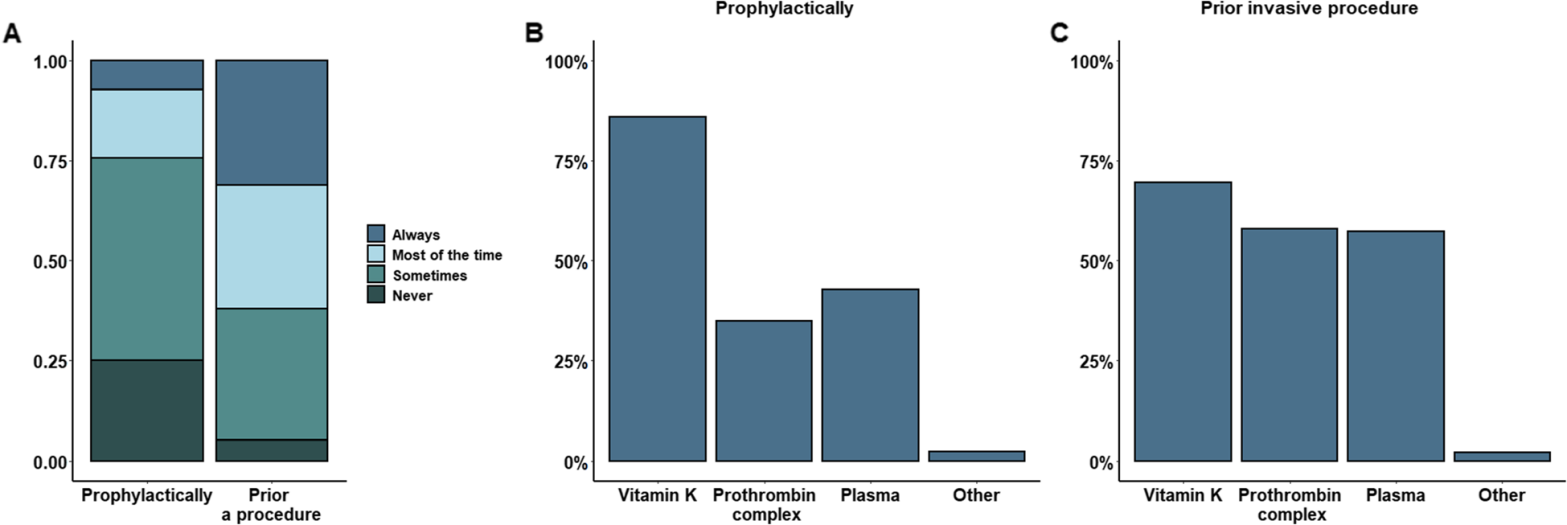
More respondents would correct a vitamin K-induced INR > 3 prior to an invasive procedure than in the absence of a procedure (**a**). Both in the absence of an invasive procedure (**b**) and prior to an invasive procedure (**c**), the majority would correct this with vitamin K

In non-bleeding patients who will not undergo an invasive procedure, an international normalized ratio (INR) > 3 is infrequently corrected. Only 7% would always correct a prolonged INR (Fig. 4a.). Vitamin K is the most commonly mentioned therapeutic agent to correct the INR in these patients (85%), followed by plasma (43%) and prothrombin complex (35%) (Fig. 4b).

More physicians would correct an INR > 3 prior to an invasive procedure compared to patients who are not undergoing an invasive procedure (*p* < 0.0001). Among the respondents, 31% would always correct a prolonged INR in this setting (Fig. 4a). Also, prior to an invasive procedure, the majority (70%) of the respondents would use vitamin K as a therapeutic option, followed by prothrombin complex (58%) and plasma (57%) (Fig. 4c).

To diagnose coagulopathy, INR/prothrombin time (99%), activated partial thromboplastin time (APTT, 97%) and fibrinogen level (94%) are widely available tests in the ICU. Viscoelastic tests are less common; only in the minority of the hospitals rotational thromboelastometry (ROTEM, 31%) or thromboelastography (TEG, 18%) is available as a diagnostic tool.

### Guideline

Among the respondents, 29% have an ICU-specific and 53% a (not ICU specific) transfusion guideline in their ICU. The availability of a guideline has a limited effect on the transfusion practice. Only for the general ICU population the presence of a (not ICU) specific transfusion guideline was associated with a lower transfusion threshold (*p* = 0.028, Additional file 1: Table S3). For other ICU subpopulations, this association with RCC transfusion practice was not present (Additional file 1: Table S2 and S3). Also, for platelet and plasma transfusion, no association was found between the presence of a guideline and transfusion practice (Additional file 1: Table S5 and S6).

### Background specialty

To investigate whether the base specialty influences transfusion practices, only the group anaesthesiology (69% of the respondents) and internal medicine (20% of the respondents) were sufficiently present to perform an additional testing. For RCC, PC and plasma transfusion, an association was found between the base specialty and transfusion practice. Overall, the base specialty in anaesthesiology was associated with a more liberal transfusion practice compared to internal medicine. With the exception of patients with ACS and patients on ECMO, physicians with the base specialty in anaesthesiology said to transfuse all subpopulations at significantly higher Hb thresholds. Furthermore, anaesthesiologists more often report the use of physiological triggers in addition to Hb levels in their decision to transfuse than internal medicine physicians (*p* = 0.02, see Additional file 1: Table S4). Also, thrombocytopenic patients are transfused at higher platelet counts prior to CVC placement (*p* = 0.002) and prior to tracheotomy (*p* = 0.007, Additional file 1: Table S7) when treated by a physician with the base specialty in anaesthesiology. For plasma transfusion, only in prophylactic transfusions a different practice was observed between these two base specialties. Physicians with a base specialty in anaesthesiology transfuse plasma prophylactically more frequently (Additional file 1: Figure S3).

### Regional differences

In all world regions, a median Hb threshold of 7 g/dL (7.7) was reported for the general ICU population. For different subpopulations, a greater variety was reported, especially in patients with ACS and traumatic brain injury and patients receiving ECMO (Additional file 1: Figure S2). In platelet transfusion, some alignment exists for prophylactic platelet transfusion with a median applied platelet count prophylactically between 15 and 20 × 10^9^ cells/L for all world regions. However, prior to an invasive procedure, more heterogeneity exists (Additional file 1: Figure S4). Prior to general surgery, the largest differences between the regions were observed; in half of the regions, the mean of the applied platelet threshold was 50 × 10^9^ cells/L while in Southern Asia and Southeastern Asia, a median platelet count of 72.5 and 100 × 10^9^ cells/L was applied, respectively. Lastly, also plasma transfusion practices differed between world regions (Additional file 1: Figure S5). In Southern Europe, only 17% would never correct a vitamin K-induced INR > 3 in the absence of an invasive procedure, whereas in Southeastern Asia, 50% would never correct this INR.

## Discussion

This is the largest survey on transfusion practice in non-bleeding critically ill patients among ICU physicians to date. The main findings of this study are (1) a high Hb threshold variation between ICU subpopulations; (2) the platelet transfusion threshold prior to invasive procedures differs greatly between and within the procedures; (3) plasma is considered by a large number of physicians in non-bleeding patients even in the absence of an invasive procedure; (4) base specialty of physicians is associated with variation in transfusion practices; and (5) worldwide, institutions lack local ICU-specific transfusion guidelines.

The reported Hb threshold for the general ICU population in this survey is in line with the finding of the TRICC study, which demonstrated the safety of a restrictive transfusion strategy in the ICU population [8]. However, when looking at different patient subpopulations, a greater variety of applied Hb thresholds was found. For the septic patients, respondents reported a significantly higher Hb threshold compared to the general population, which deviates from current evidence supporting a restrictive transfusion strategy also in septic patients [11]. For patients with ACS, the higher preferred Hb threshold of 9 g/dL (8–9.6) is in accordance with the transfusion guideline from the National Institute for Health and Care Excellence (NICE, 2018), in which a Hb threshold (8–10 g/dL) for patients with symptomatic coronary disease is advised. Also, patients with traumatic brain injury were transfused at higher Hb thresholds since these patients may be more sensitive to anaemia-induced cerebral hypoxia. However, evidence to justify this more liberal transfusion practice is limited. Multiple large RCTs are currently studying whether these patients benefit from a liberal transfusion strategy (ClinicalTrials.gov, NCT02968654 and NCT02981407).

This survey also showed a high variety of preferred Hb thresholds for patients with ARDS and patients on ECMO. Since the evidence for these subpopulations is limited, it is expected to observe a high heterogeneity in transfusion practice. For ARDS patients, it is hypothesized that the hypoxaemia should be compensated by increasing the oxygen-carrying capacity of the circulating blood by transfusing at higher Hb thresholds. However, there is no solid evidence to support this practice, and the downside of allogenic blood transfusion is not taken into account in this reasoning.

The applied platelet threshold differed between patients with and without an upcoming invasive procedure. The majority of the respondents (72%) would transfuse non-bleeding critically ill patients at a platelet count of ≤ 20 × 10^9^ cells/L. The potential harm of platelet transfusion is supported by two recent RCTs, in which it was shown that prophylactic platelet transfusion might be particularly harmful in neonates [16] and in patients with a cerebrovascular accident [17]. These studies cannot be directly translated to the non-bleeding critically ill adult patients, but they do show that platelet transfusion is not an intervention without a risk. Prior to invasive procedures, physicians transfuse platelets at higher platelet counts, while the evidence for this is limited. A meta-analysis has shown that complications prior to CVC placement in patients with coagulopathy, including thrombocytopenia and prolonged INR and APTT, are rare [18]. Thus, the need for any platelet transfusion prior to this procedure is questionable. A large RCT studying the need for platelet transfusion prior to CVC placement in severely thrombocytopenic patients is now recruiting [19].

Multiple RCTs have failed to demonstrate beneficial effects of prophylactic plasma transfusion prior to an invasive procedure in critically ill patients with a prolonged INR [12, 20, 21]. The finding of this survey that 57% and 43% of physicians would consider transfusing plasma to correct the prolonged INR in patients who used vitamin K antagonists prior to an invasive procedure or without a planned invasive procedure, respectively, is striking in the absence of evidence for this practice.

To our knowledge, the influence of base specialty of intensivists on transfusion practice has not been studied before. Our survey showed that doctors with a base specialty in anaesthesiology transfuse more liberally than those with internal medicine as a base specialty. It might be that doctors with an internal medicine background are more aware of the harmful side effects of blood products; alternatively, anaesthesiologists may tend to treat patients at a higher risk of bleeding, and these practices spill over into the ICU.

The strength of this survey is the large number of respondents. However, both the anonymous character of this survey and the origin of the respondents might have introduced a selection bias and limits therefore the worldwide generalizability of our findings. Theoretically, it is possible that multiple respondents are employed in the same hospital; however, also within hospitals heterogeneity in transfusion practice may exist. Furthermore, the number of respondents who did receive this survey but did not fill it in is unknown. We cannot exclude that non-responders transfuse differently than the responders of this survey. It may be possible that physicians with more interest in transfusion practice and thus with more awareness of the possible side effects of transfusion are over presented in this survey. In addition, due to the study design, it was not appropriate to perform multivariable analysis. As a result, it was not possible to exclude the presence of confounding variables on the observed significant associations. And finally, as with any clinical practice survey, the reported transfusion practices might differ from actual transfusion practices. Ideally, these results are confirmed in a prospective cohort study.

## Conclusion

In conclusion, in the general non-bleeding ICU population, the reported RCC transfusion practice was rather restrictive; however, in certain subpopulations including the critically ill with septic shock, higher applied Hb thresholds were reported, which deviates from the current evidence. For other subpopulations such as patients with ARDS and patients on ECMO, well-powered RCTs are needed. In addition, optimal platelet thresholds are currently controversial and more awareness is necessary for the correct indications of plasma transfusion in non-bleeding patients. Finally, a local transfusion guideline for critically ill patients is lacking in the majority of ICUs worldwide.

## Additional file

Additional file 1: Supplements TRACE survey. (DOCX 228 kb)

## Data Availability

The datasets used and analysed during the current study are available from the corresponding author on reasonable request.
