# Supplementary material for: Transfusion practice in the non-bleeding critically ill: an international online survey—the TRACE survey
Source: Crit Care. 2019 Sep 11;23:309. doi: 10.1186/s13054-019-2591-6 (PMC6737617; doi:10.1186/s13054-019-2591-6)
Supplement: Supplementary file 1 — Supplements TRACE survey. (DOCX 228 kb) [file 13054_2019_2591_MOESM1_ESM.docx]

**Additional file 1**

Figures


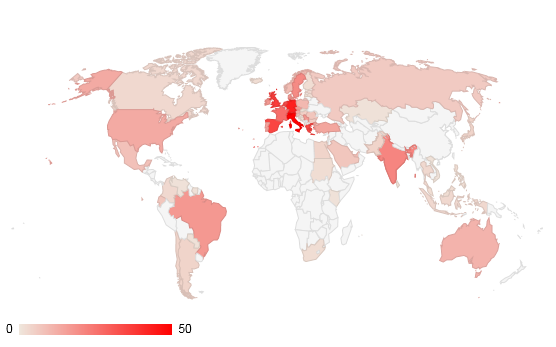


**Figure S1** Origin of respondents


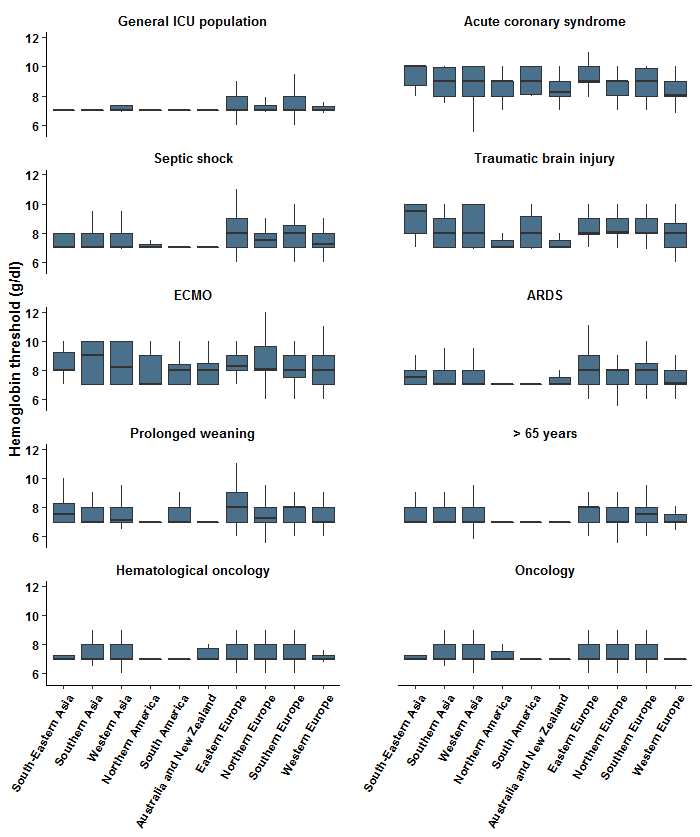


**Figure S2.** Patients with acute coronary syndrome were transfused at highest Hb levels in South Eastern Asia with a median Hb level of 10 g/dL (8.75-10) while in Western Europe the median reported transfusion threshold was 8.1 g/dL (8-8.1). For patients with traumatic brain injury also respondents from South Eastern Asia reported the highest Hb levels as transfusion thresholds of 9.5 g/dL (8-9.5) and lowest Hb levels were reported in Northern America with a median Hb of 7 g/dL (7-7). In Southern Asia the highest Hb thresholds for patients receiving ECMO were reported with 9 g/dL (7-9), while the lowest Hb levels were reported by respondents working in Northern America with 7 g/dL (7-7).


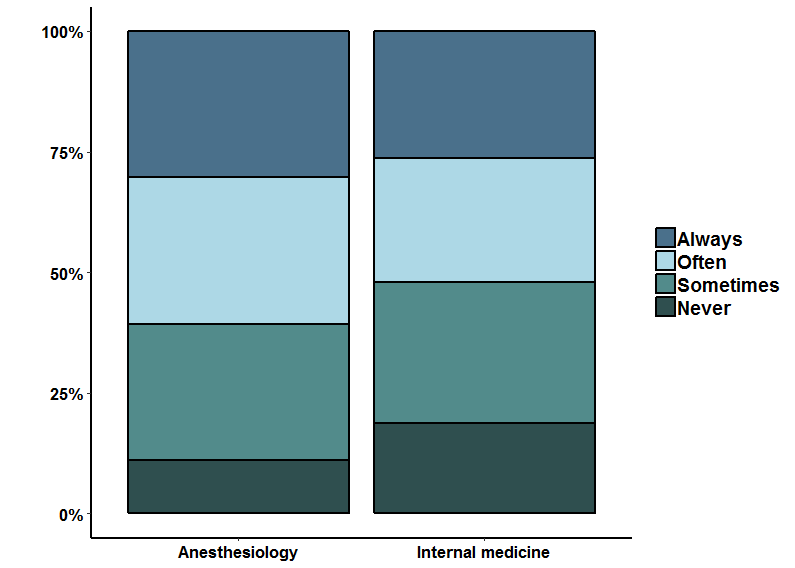


**Figure S3.** The use of a physiological transfusion trigger in addition to a Hb threshold. Significant differences were observed for anaesthesiology versus internal medicine (p = 0.02).


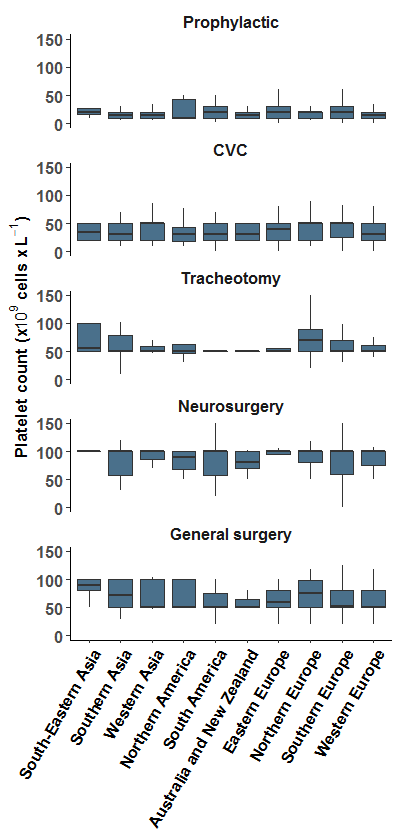


**Figure S4.** Regional differences of applied platelet thresholds prophylactically without any planned invasive procedure and prophylactically prior to different procedures.


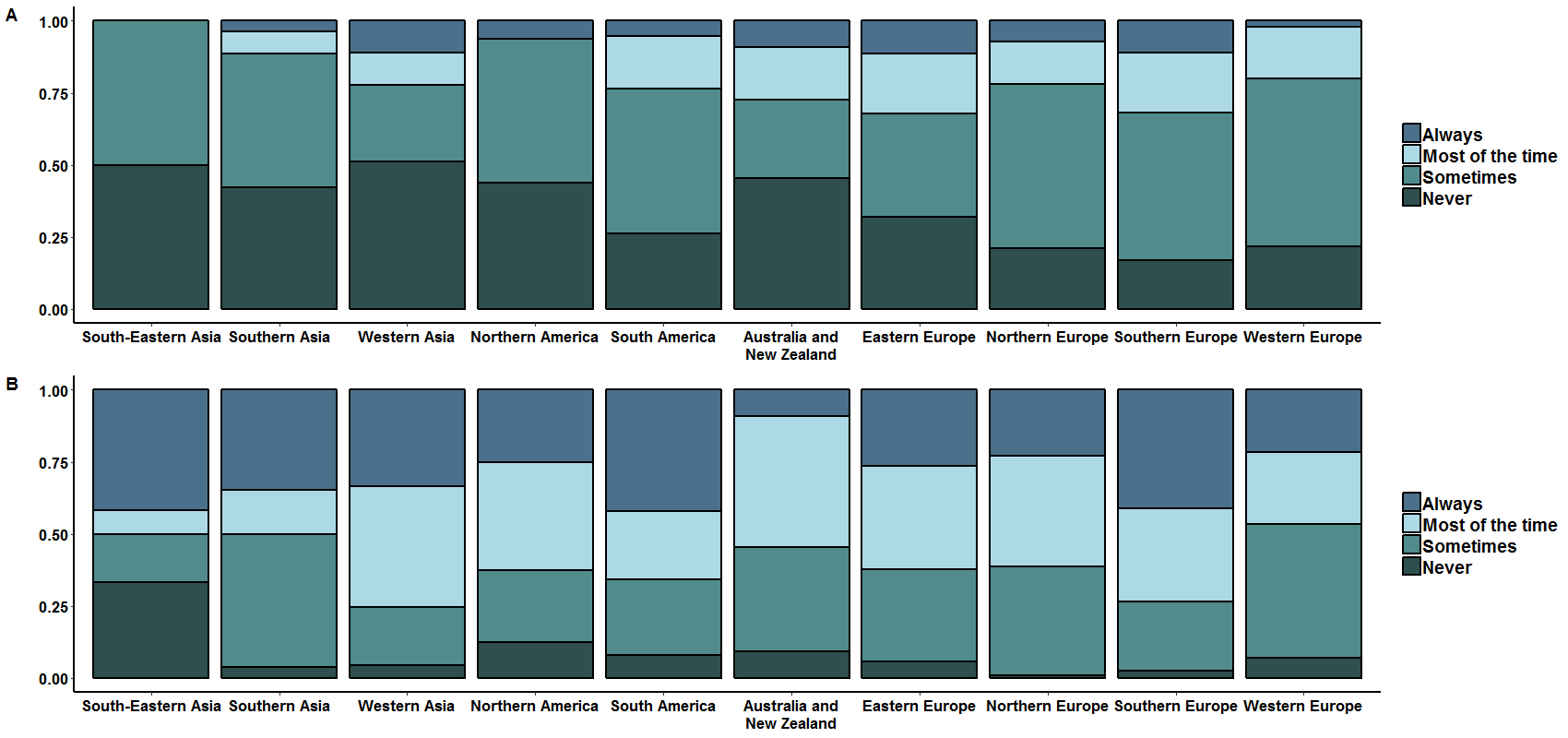


**Figure S5.** Regional differences of the correction of a vitamin K induced INR >3 (A) prophylactically in the absence of an invasive procedure and (B) prior to an invasive procedure.


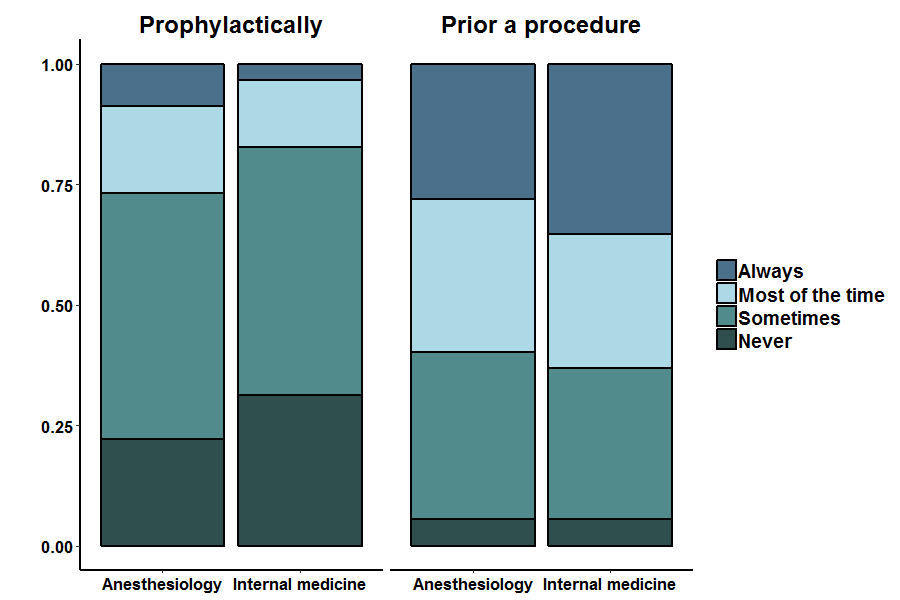


**Figure S6.**

Effect of base specialty of ICU doctor on the correction of a vitamin K induced INR >3 (A) prophylactically in the absent of an invasive procedure (p= 0.0272) and (B) prior to an invasive procedure (p=0.03983).

**Table S1**

| **World region** | **n =** |
| --- | --- |
| Australia and New Zealand | 11 |
| Caribbean | 1 |
| Central America | 8 |
| Central Asia | 1 |
| Eastern Africa | 2 |
| Eastern Asia | 4 |
| Eastern Europe | 53 |
| Northern Africa | 6 |
| Northern America | 16 |
| Northern Europe | 109 |
| South-Eastern Asia | 12 |
| South America | 38 |
| Southern Africa | 2 |
| Southern Asia | 26 |
| Southern Europe | 197 |
| Western Asia | 45 |
| Western Europe | 184 |

**Table S2**

|  | **ICU specific guideline** | |  |
| --- | --- | --- | --- |
|  | **Available** | **Not available** | **p value** |
| **General ICU population** | 7(7-7.5) | 7(7-7.5) | 0.1341 |
| **Acute coronary syndrome** | 9(8-9.7) | 9(8-9.6) | 0.1009 |
| **Septic shock** | 7.3(7-8) | 7.5(7-8) | 0.389 |
| **Traumatic brain injury** | 8(7-9) | 8(7-9) | 0.9795 |
| **ECMO** | 8(7-9) | 8(7.5-9) | 0.2455 |
| **ARDS** | 7(7-8) | 7.5(7-8) | 0.0517 |
| **Prolonged weaning** | 7.5(7-8) | 7.4(7-8) | 0.9892 |
| **Age ≥65 years** | 7(7-8) | 7(7-8) | 0.6015 |
| **Haematological oncology** | 7(7-8) | 7(7-8) | 0.2184 |
| **Oncology** | 7(7-8) | 7(7-8) | 0.8695 |

**Table S3**

|  | **(not-ICU specific) Guideline** | |  |
| --- | --- | --- | --- |
|  | **Available** | **Not available** | **p value** |
| **General ICU population** | 7(7-7.5) | 7(7-7.9) | 0.0283 |
| **Acute coronary syndrome** | 9(8-9.7) | 9(8-9) | 0.8161 |
| **Septic shock** | 7.3(7-8) | 7.5(7-8) | 0.2662 |
| **Traumatic brain injury** | 8(7-9) | 8(7-9) | 0.783 |
| **ECMO** | 8(7-9) | 8(7-9) | 0.2794 |
| **ARDS** | 7(7-8) | 7.5(7-8) | 0.262 |
| **Prolonged weaning** | 7.1(7-8) | 7.5(7-8) | 0.1283 |
| **Age ≥65 years** | 7(7-8) | 7(7-8) | 0.0649 |
| **Haematological oncology** | 7(7-8) | 7(7-8) | 0.3209 |
| **Oncology** | 7(7-8) | 7(7-8) | 0.191 |

**Table S4**

|  | **Main specialty** | | |  |
| --- | --- | --- | --- | --- |
|  | **Anaesthesiology** | **Internal medicine** | **p value** | |
| **General ICU population** | 7(7-8) | 7(7-7) | | 0.0452 |
| **Acute coronary syndrome** | 9(8-9.7) | 9(8-9.9) | | 0.3662 |
| **Septic shock** | 8(7-8) | 7(7-8) | | < 0.001 |
| **Traumatic brain injury** | 8(7.8-9) | 8(7-9) | | 0.036 |
| **ECMO** | 8(7.7-9) | 8(7-9.5) | | 0.5545 |
| **ARDS** | 8(7-8.2) | 7(7-8) | | < 0.001 |
| **Prolonged weaning** | 8(7-8) | 7(7-8) | | < 0.001 |
| **Age ≥65 years** | 7.2(7-8) | 7(7-7.7) | | 0.0003 |
| **Haematological oncology** | 7(7-8) | 7(7-7.4) | | 0.0224 |
| **Oncology** | 7(7-8) | 7(7-7.2) | | 0.0459 |

**Table S5**

|  | **ICU specific transfusion guideline** | |  |
| --- | --- | --- | --- |
|  | **Available** | **Not available** | **p value** |
| **Prophylactic** | 20(10-20) | 20(10-30) | 0.2827 |
| **Prior to CVC placement** | 30(20-50) | 45(20-50) | 0.1299 |
| **Prior to tracheotomy** | 50(50-70) | 50(50-71) | 0.7226 |
| **Prior to general surgery** | 50.5(50-93) | 50(50-80) | 0.8616 |
| **Prior to neurosurgery** | 100(67.5-100) | 100(75-100) | 0.3684 |

**Table S6**

|  | **(not-ICU specific) transfusion guideline** | |  |
| --- | --- | --- | --- |
|  | **Available** | **Not available** | **p value** |
| **Prophylactic** | 20(10-25) | 20(10-30) | 0.8973 |
| **Prior to CVC placement** | 45(20-50) | 40(20-50) | 0.5185 |
| **Prior to tracheotomy** | 50(50-71) | 50(50-70) | 0.1976 |
| **Prior to general surgery** | 50(50-80) | 53(50-90) | 0.4122 |
| **Prior to neurosurgery** | 100(70-100) | 100(75-100) | 0.1442 |

**Table S7**

|  | | **Main specialty** | |  |
| --- | --- | --- | --- | --- |
|  | **Anaesthesiology**  **Platelet count** | | **Internal medicine**  **Platelet count** | **p value** |
| **Prophylactic** | 20(10-30) | | 10(10-20) | 0.0052 |
| **Prior to CVC placement** | 48.5(20-50) | | 30(20-50) | 0.0023 |
| **Prior to tracheotomy** | 50(50-80) | | 50(50-51) | 0.0069 |
| **Prior to general surgery** | 51(50-80) | | 50(50-81) | 0.3439 |
| **Prior to neurosurgery** | 100(75-100) | | 100(80-100) | 0.9409 |

**Appendix 1: Overview collaborating societies**

- Australian and New Zealand Intensive Care Society (ANZICS)
- Czech Society of Anaesthesiology, Resuscitation & Intensive Care (CSARIM)
- Hellenic Society of Intensive Care (HSIC)
- Societa Italiana di Anestesia Analgesia Rianimazione e Terapia Intensiva (SIAARTI)
- Intensive Care Society of Ireland (ICSI)
- Lebanese Critical Care Society (LCCS)
- Nederlandse Vereniging voor Intensive Care (NVIC)
- Serbian Association of Anaesthesiologists & Intensivits (SAAI)
- Slovenian Society of Intensive Medicine (SSIM)
- Swedish Society of Anaesthesiology & Intensive Care Medicine (SFAI)
- Swiss Society of Intensive Care Medicine (SGI-SSICM)
- Turkish Society of Anesthesiology and Reanimation (TARD)
- Intensive Care Society (ICS, United Kindom)

**Appendix 2: Static version questionnaire**

**Demographics:**

1. In which country do you work?
2. What is your intensive care certification level
   1. Intensivist
   2. Resident, specialist in training
   3. Specialist non intensivist practising ICU
   4. Nurse
   5. Student
   6. Other, please specify
3. What is your primary medical specialty
   1. Anaesthesiology
   2. Cardiology
   3. Internal medicine
   4. Neurology
   5. Paediatrics
   6. Pulmonology
   7. Surgery
   8. Other, please specify
4. Type of intensive care unit (ICU)
   1. Medical ICU
   2. Surgical ICU
   3. Mixed ICU
   4. Other, please specify
5. Number of ICU beds
   1. <10
   2. 10-15
   3. 16-20
   4. >20
6. Annual number of patients treated in the ICU
   1. <500
   2. 500-1000
   3. 1001-1500
   4. 1501-2000
   5. >2000
7. Type of institution
   1. University hospital
   2. University affiliated hospital
   3. Non-university public hospital
   4. Private hospital
   5. Other, please specify
8. Do you have a transfusion protocol in your hospital?
   1. Yes
   2. No
   3. I don’t know
   4. Other, please specify
9. Do you have a transfusion protocol specific for the intensive care unit in your hospital?
   1. Yes
   2. No
   3. I don’t know
   4. Other, please specify

**Red blood cell transfusion**

1. Which unit do you use to measure hemoglobin levels?
   1. g/dL
   2. g/L (=mg/ml)
   3. mmol/L
2. What is your overall threshold for blood transfusion in a general population of anemic critically ill patients?
3. What is your threshold for blood transfusion in anemic critically ill patients with acute coronary syndrome?
4. What is your threshold for blood transfusion in anemic critically ill patients with septic shock?
5. What is your threshold for blood transfusion in anemic critically ill patients with traumatic brain injury?
6. What is your threshold for blood transfusion in anemic critically ill patients receiving ECMO?
7. What is your threshold for blood transfusion in anemic critically ill patients with ARDS?
8. What is your threshold for blood transfusion in anemic critically ill patients with prolonged weaning from mechanical ventilation?
9. What is your threshold for blood transfusion in anemic critically ill patients >65 years?
10. What is your threshold for blood transfusion in anemic critically ill patients with a hematologic malignancy?
11. What is your threshold for blood transfusion in anemic critically ill oncology patients?
12. Do you check hemoglobin levels after transfusion of one unit red cell concentrates before transfusing a second unit in non-bleeding critically ill patients?
    1. Always
    2. Most of the time
    3. Sometimes
    4. Never
13. Do you also use physiological transfusion triggers (e.g. tachycardia) in addition to a hemoglobin threshold?
    1. Always
    2. Often
    3. Sometimes
    4. Never
14. Which physiological transfusion triggers do you use in non-bleeding patients? (multiple answers possible)
    1. Tachycardia
    2. Hypotension
    3. Arrhythmia
    4. Significant ECG changes
    5. SvO_2_ (mixed venous saturation of oxygen) < 65 %
    6. ScvO_2_ (central venous oxygen saturation) < 65 %
    7. Lactate >2 mmol/L
    8. Acidosis
    9. Other, please specify
15. How would you rank the following triggers of importance to you? (1 means most important)
    1. All triggers ticked by respondent in previous question are mentioned.
16. Which of the following treatment options do you use to prevent transfusion of red cell concentrates in anemic critically ill patients with iron deficiency? (multiple answers possible)
    1. Iron suppletion
    2. Erythropoietin (epo) suppletion
    3. Iron suppletion in combination with erythropoietin (epo)
    4. None of the above
    5. Other, please specify
17. Which measures are available in your ICU to minimize the amount of red cell concentrate transfusion? (multiple answers possible)
    1. Closed loop sampling
    2. Computer decision making program
    3. Microtube sampling
    4. None
    5. Other, please specify

**Platelets**

1. What is your threshold for prophylactic platelet transfusion in thrombocytopenic non-bleeding patients on the ICU (thus not prior to an invasive procedure)? (10^9 cells/L)
2. What is your threshold for platelet transfusion in non-bleeding thrombocytopenic patients prior to placing a central line on the ICU? (10^9 cells/L)
3. What is your threshold for platelet transfusion in critically ill non-bleeding thrombocytopenic patients prior to a tracheotomy? (10^9 cells/L)
4. What is your threshold for platelet transfusion in thrombocytopenic non-bleeding critically ill patients prior to neurosurgery? (10^9 cells/L)
5. What is your threshold for platelet transfusion in thrombocytopenic non-bleeding critically ill patients on the ICU prior to general surgery? (10^9 cells/L)
6. Do you check thrombocyte count after transfusion of one unit thrombocyte concentrate (1 unit is approx. 300 x 10^9 platelets) before transfusing a second unit in non-bleeding critically ill patients?
   1. Always
   2. Most of the time
   3. Sometimes
   4. Never

**Plasma**

1. Which coagulation tests are available as standard care on your ICU? (multiple answers possible)
   1. PT/INR
   2. aPTT
   3. Fibrinogen
   4. Rotational thromboelastometry (ROTEM)
   5. Thromboelastography (TEG)
   6. Other, please specify
2. Do you check the INR after transfusion of one unit of plasma before transfusing a second unit in a non-bleeding patient with plasmatic coagulopathy (INR>3.0) who used vitamin K antagonists?
   1. Always
   2. Most of the time
   3. Sometimes
   4. Never
   5. Not applicable
3. How do you correct a plasmatic coagulopathy (INR>3.0) prophylactically in non-bleeding patients who used vitamin K antagonists on the ICU? (multiple answers possible)
   1. Vitamin K
   2. Cofact (prothrombin complex)
   3. Plasma
   4. Other, please specify
4. Do you correct a plasmatic coagulopathy (INR >3) in non-bleeding critically ill patients on the ICU prior to an invasive procedure (e.g. placing a central line) who used vitamin K antagonists?
   1. Always
   2. Most of the time
   3. Sometimes
   4. Never
5. How do you treat plasmatic coagulopathy (INR >3.0) prior to an invasive procedure in non-bleeding patients who used vitamin K antagonists on the ICU? (multiple answers possible)
   1. Vitamin K
   2. Cofact (prothrombin complex)
   3. Plasma
   4. Other, please specify
6. Do you check the INR after transfusion of one unit of plasma before transfusing a second unit in a non-bleeding patient with plasmatic coagulopathy (INR >3.0) prior to an invasive procedure on the ICU who used vitamin K antagonists?
   1. Always
   2. Most of the time
   3. Sometimes
   4. Never
   5. Not applicable
7. If you decide to transfuse a non-bleeding critically ill patient, what type of plasma do you use? (multiple answers possible)
   1. Pooled plasma (e.g. Omniplasma)
   2. FFP (fresh frozen plasma)
   3. Other (please specify)
